# Chondroitinase Versus Papain Digestion Leads to Different Outcome for In Vitro Simulation of Degenerated Discs

**DOI:** 10.1002/jsp2.70164

**Published:** 2026-03-01

**Authors:** Jan Ulrich Jansen, Graciosa Quelhas Teixeira, Elias Salzer, Andrea Vernengo, Sibylle Grad, Keita Ito, Cornelia Neidlinger‐Wilke, Hans‐Joachim Wilke

**Affiliations:** ^1^ Institute of Orthopaedic Research and Biomechanics Ulm University Medical Centre Ulm Germany; ^2^ Orthopaedic Biomechanics, Department of Biomedical Engineering Eindhoven University of Technology Eindhoven the Netherlands; ^3^ Department of Chemical Engineering, Henry M. Rowan College of Engineering Rowan University Glassboro New Jersey USA; ^4^ AO Research Institute Davos Davos Switzerland

**Keywords:** biomechanics, bovine tail, chondroitinase ABC, disc height, organ culture model, range of motion

## Abstract

**Background:**

Biomaterials play an increasing role in intervertebral disc regeneration and require preclinical testing, typically performed using organ culture and in vitro models. Native human discs are limited, and animal models often fail to mimic human disc degeneration. Thus, enzymes like chondroitinase ABC (chABC) and papain are used to simulate degenerative tissue changes and enable biomaterial injection. In previous work, we characterized the biomechanical and morphological effects of papain, which forms cavities in the disc. In contrast, chABC does not form cavities, but its biomechanical effects remain insufficiently characterized. This study aims to evaluate the macroscopic and biomechanical effects of chABC—specifically, range of motion (ROM), neutral zone (NZ), and disc height—in a bovine organ culture model, and assess the distribution of an injected hydrogel, comparing the results to published papain data.

**Methods:**

Four groups of fresh bovine tail segments were prepared (*n* ≥ 10) and three received injections of chABC, papain, or PBS, followed by 7 days of culture. For papain and PBS, published data were supplemented with new specimens. Complex simulated physiological loading was applied to diminish disc swelling. The maximum volume of a serum‐albumin‐hydrogel was injected into all four groups. ROM, NZ, and disc height were measured before and after enzyme treatment, loading, and injection. Post‐injection, microCT scans visualized material distribution within the discs.

**Results:**

ChABC increased ROM by up to 92.1%, NZ by up to 79.4%, and decreased disc height by 2.1 mm. Hydrogel injection decreased ROM and NZ but increased disc height in all groups while enzyme treatments allowed more hydrogel injection (0.6 mL for chABC). Exemplary scans showed cloud‐like hydrogel spread for chABC and a round‐shaped degradation defect for papain.

**Conclusions:**

The findings indicate that chABC better simulates disc degeneration, whereas papain better models nucleotomies, and both enzymes preserve annulus integrity—providing valuable models for biomechanical testing.

## Graphical abstract

Chondroitinase ABC and papain similarly alter disc biomechanics and increase injectable volume, but produce fundamentally different structural morphologies. Papain induces cavities, whereas chondroitinase preserves tissue integrity. Hydrogel distributions differ—cloud‐like for chABC, monolithic for papain—supporting chondroitinase as a degeneration and papain as a nucleotomy model.

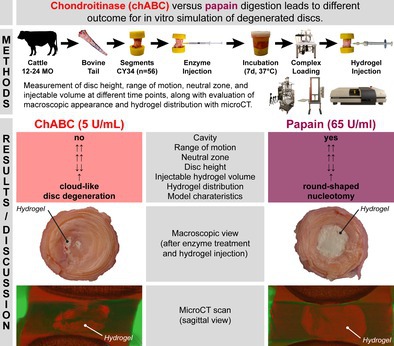

## 1 Introduction

Low back pain (LBP) is the leading cause of disability and morbidity worldwide, with an estimated 619 million people affected globally in 2020, highlighting its substantial and ongoing economic burden [1, 2, 3]. LBP is predominantly associated with intervertebral disc (IVD) degeneration [4, 5, 6, 7], which is characterized by limited nutrient supply, hypoxia, increased cell death, breakdown of the extracellular matrix, and decreased intradiscal pressure in the inner region of the IVD, the nucleus pulposus (NP) [8, 9, 10, 11]. As degeneration progresses, the IVD's ability to distribute mechanical loads diminishes [12], resulting in excessive stress concentrations and increased shear stress in the annulus fibrosus (AF), the outer region of the IVD. These changes may lead to disc bulging and fissure formation [11, 12, 13, 14]. Novel therapeutic approaches aim to halt or even reverse disc degeneration [15, 16, 17, 18], typically requiring direct intradiscal injections into the NP. Among these, injectable biomaterials—particularly hydrogels—are showing promising results in vitro, creating a need for suitable preclinical models to evaluate their performance [17, 19].

**FIGURE 1:**
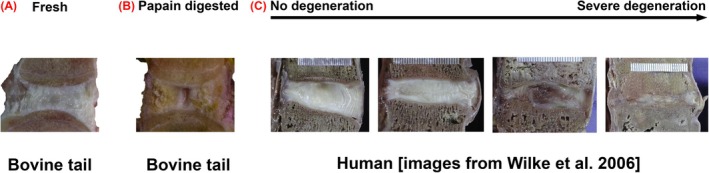
Comparison of disc degeneration in bovine tail models artificially provoked by papain versus human disc degeneration. (A) Midsagittal view of bovine tail specimen in the intact condition. (B) Midsagittal view of bovine tail specimen after 7 days of 65 U/mL papain treatment. (C) Exemplary midsagittal view of four grades of disc degeneration in human lumbar discs (from left to right): Grade 0—no degeneration, grade 1—mild degeneration, grade 2—moderate degeneration, and grade 3—severe degeneration [22].

Various animal models have been developed for biomechanical and preclinical testing [19]. Functional spinal units (FSUs) from the bovine tail have become widely used due to their easy availability from slaughterhouses, tissue viability, and similarities to human discs in terms of matrix composition [19, 20, 21]. However, IVDs from young animals typically exhibit minimal natural degeneration and high swelling‐pressure, limiting the volume of biomaterials that can be injected (Figure 1A) [23, 24, 25]. To overcome this, enzymes are often used to artificially provoke degenerative changes by digesting IVD tissue, apart from mechanical damage and proinflammatory cytokines [24]. With a peak in the 1980s and 1990s for chymopapain, the enzymes papain, chondroitinase ABC (chABC) and collagenase II have been clinically used for herniated disc treatment, that is, chemonucleolysis [26, 27]. Recently, these enzymes have been used to simulate disc degeneration in vitro [23, 24, 26, 27, 28, 29, 30]. Papain and collagenase II are characterized by the formation of cavities (Figure 1B) that allow the injection of large amounts of biomaterial (e.g., 0.73 mL hydrogel per disc with papain) [23, 28]. At a concentration of 65 U/mL, papain also significantly increases range of motion (ROM, +109.5%) and neutral zone (NZ, +210.9%), while reducing disc height (−1.96 mm) [23]. In contrast, chABC acts more selectively, cleaving sulfated glycosaminoglycan (GAG) chains into disaccharides without forming cavities. By cleaving chondroitin and dermatan sulfates chains and allowing their fragments to leave the disc, chABC reduces the NP's water‐binding capacity and intradiscal pressure [31, 32, 33]. At the tissue level, chABC (5 U/mL, 7 days) is known to decrease the equilibrium stress (a stiffness measure) about sevenfold and induces degeneration equivalent to Thompson grade IV by day 3 [33, 34]. These characteristics suggest that chABC may more closely mimic physiological disc degeneration compared to papain [23, 24, 35], which is further supported by morphological similarities to early degenerated human discs (Figure 1B vs. Figure 1C). A key advantage of enzymatic models is that, through targeted injections into the NP, they preserve the structural integrity of the AF. Wilke et al. demonstrated that an intact AF plays a crucial role in assessing the risk of biomaterial extrusion when using bovine lumbar FSUs [25]. This makes enzymatic models valuable for preclinical testing of extrusion risk at the segmental level.

However, biomechanical data on chABC treatment in vitro—particularly in bovine tail FSUs—are lacking, making it difficult to compare the effects of chABC with papain. Takahashi et al. have observed reduced disc height in rabbits after in vivo treatment with 4 U chABC for up to 10 days [31]. Sugimura et al. compared chABC with papain in monkeys in vivo and found greater disc height loss with papain, but less toxicity and more specific GAG digestion with chABC [36]. Lu et al. observed an increase of ROM and NZ for canine specimens treated with chABC (up to 5 U for 1 week) in vivo [37]. Despite this, there is a need to better understand the biomechanical effects of chABC in bovine tail FSUs and to compare them with papain.

The aim of this study was to assess the effects of chABC injection on macroscopic appearance, ROM, NZ, disc height, and hydrogel injectability using a bovine tail organ culture model. These outcomes after hydrogel injection, as well as differences in macroscopic appearance, biomechanical response, and hydrogel distribution were compared with those of papain and controls (sham and fresh) to determine the suitability of enzyme treatment for biomaterial evaluations. Comparisons between enzyme treatments alone are presented in the discussion section, as the results for papain and sham were already reported in an earlier publication [23].

## 2 Materials and Methods

**FIGURE 2:**
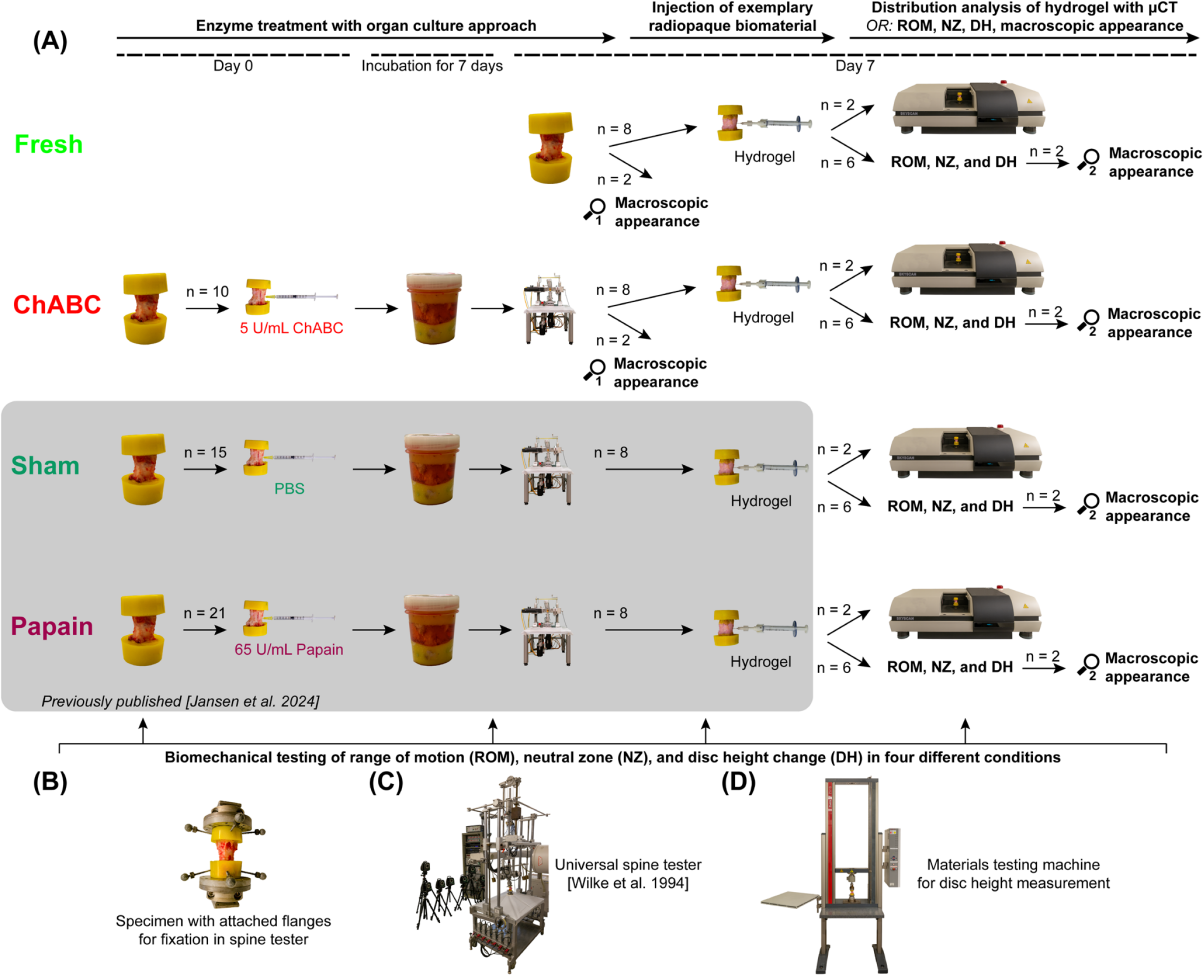
(A) Two groups with enzymatic digestion (chABC and papain) and two control groups (fresh and sham) were used to compare the effects of enzyme treatment on macroscopic appearance, range of motion (ROM), neutral zone (NZ), disc height change (DH), injectable volume, and hydrogel distribution. For comparison, data from a previous publication for the sham and papain groups are presented in Section 4 [23]. Hydrogel distribution was assessed using radiopaque hydrogel and micro‐CT scans. ROM, NZ, and DH were measured in four different conditions: intact, after incubation, after loading, and after injection of hydrogel. (B) Specimens were fixated in biomechanical testing machines via flanges that could be screwed onto the PMMA embedding of the specimens. (C) For determining ROM and NZ, a universal spine tester [39] was used applying pure moments of ±1 Nm with a speed of 1°/s [39, 40]. (D) DH was measured under a standardized load of 30 N with a materials testing machine.

A new organ culture approach has been developed to test the biomechanical effects of enzymatic digestion on bovine tail segments in a sequential approach [38] (previously performed for papain and a sham group by Jansen et al. [23]). In this study, the same method was used to investigate the effects of chABC on the macroscopic appearance, range of motion (ROM), neutral zone (NZ), and disc height (Figure 2A). Furthermore, as an exemplary biomaterial, a serum albumin hydrogel (Albugel, Section 2.6) was injected into IVDs of four different test groups (fresh, chABC, sham, papain) to obtain the biomechanical effects, the maximal injectable volume, as well as the distribution pattern of injected hydrogels for these four different groups.

### 2.1 Specimens

A total of 56 bovine mono‐segmental tail motion segments from 56 male cattle (12–24 months old, segment CY3/4, in combination with Jansen et al. [23]) were obtained from a local slaughterhouse (Ulmer Fleisch, Ulm, Germany). No ethical approval was required. Prior to preparation, the specimens were screened for signs of anatomic anomalies, growth abnormalities, vertebral fractures, and degenerative changes using anterior–posterior X‐ray (DR Panel PIXX1417, PIXXGEN Corporation, Gyeonggi‐do, Korea). After removing muscles and ligaments, the segments of level CY3/4 were cut out through the middle of the adjacent vertebra CY3 and CY4 and embedded in Poly(methyl methacrylate) (PMMA, Technovit 3040, Kulzer GmbH, Hanau, Germany) cranially and caudally. The segments were blindly assigned to four different groups (fresh, chABC, sham, and papain) (*n* ≥ 10/group, Figure 2A). For biomechanical testing, flanges were screwed on the hardened PMMA blocks of each functional spinal unit (Figure 2B).

### 2.2 Experimental Design

In the intact condition, the ROM, NZ, and disc height of all specimens were measured (day 7 for group fresh; day 0 for all other groups) (Figure 2A). IVDs of the groups chABC, papain, or sham were treated with enzymes or PBS, respectively, and incubated for 7 days in cell culture medium (described in Section 2.3). On day 7, the incubation was terminated and the specimens (except group fresh, freshly harvested from different animals on day 7) were loaded with a complex loading protocol to compensate for the fluid uptake during incubation [23]. Specimens were dissected for macroscopic appearance no. 1 (see number next to magnifying glass symbol in Figure 2). An exemplary radiopaque hydrogel was injected into eight specimens per group to determine the maximal injectable volume. Afterwards, the specimens were either used to analyze the hydrogel distribution with microCT (*n* = 2) or used to determine biomechanical effects from the injected hydrogel and macroscopic appearance no. 2 (*n* = 6). As “biomechanical effects”, ROM, NZ, and disc height were measured in every condition (Section 2.5 and Figure 2).

### 2.3 Enzymatic Digestion

Lyophilized chABC (C2905, Sigma‐Aldrich, St. Louis, MO, USA) was dissolved and stored in an aqueous solution with 0.01 mass‐% bovine serum albumin (BSA, A4919, Sigma‐Aldrich), with a ratio of 60 units chABC per 1 mL. This stock solution was mixed shortly prior to the injection with a 50 mM tris(hydroxymethyl)aminomethane buffer (T1503, Sigma‐Aldrich) of pH 8.0 containing 60 mM sodium acetate (S2889, Sigma‐Aldrich) and 0.02 mass‐% BSA, resulting in a final chABC concentration of 5 U/mL. A phosphate buffered solution with 65 U/mL papain and pH 6.4 was used for the papain group and PBS alone for group sham [23]. Data for the papain and sham group were published using the same experimental design and conditions, and are included here for direct comparison with chABC. As described in a previous publication of our group [23], the FSUs embedded in PMMA were cleaned and disinfected and then the enzyme solution was injected into the center of the discs with a needle size of 30 G under sterile conditions. Needle positioning was based on practical experience, with initial verification by X‐ray during method development. For all three treatments (chABC, sham, papain), the same standardized amount of 200 μL was injected; FSUs of the group fresh were only embedded in PMMA at day 7 and then tested.

After injection, the FSUs of groups chABC, sham, and papain were incubated under a free‐swelling condition at 6% O_2_ and 37°C for 7 days using 60 mL high‐glucose Dulbecco's Modified Eagle Medium (Gibco, Thermo Fisher Scientific Inc., Waltham, MA, USA) per specimen, supplemented with 5% fetal bovine serum (Sigma‐Aldrich), 1% penicillin/streptomycin, 0.5% amphotericin B, 1% non‐essential amino acids (Gibco), and 1.5% 5 mol/L NaCl/0.4 mol/L KCl solution to adjust the osmolarity of the medium to 400 mOsm. The medium was changed on day 3. Incubation was realized using sterile containers with a not fully closed screw cap (100 mL, Polyethylene, 75.562.105, Sarstedt AG & Co. KG, Nümbrecht, Germany) [23].

### 2.4 Complex Loading to Compensate Free‐Swelling

All specimens in the chABC, sham, and papain groups underwent complex loading with identical parameters to counteract disc swelling due to incubation. A custom‐built dynamic disc‐loading simulator applied cyclic axial compression ranging from 50 to 450 N at 3 Hz [23, 41, 42]. This was combined with a rotation in FE and left–right LB, both for ±10°, acting as out‐of‐phase sine and cosine waves at 1 Hz [23, 41, 42]. Following incubation, the loading was applied for 1 h, corresponding to 2700 cycles at 1 Hz and short intervals to re‐adjust the compression force baseline to 150 N [23]. The magnitude of the complex loading was selected to compensate for the stiffening mechanical behavior caused by fluid uptake during incubation in the cell culture medium, aiming to restore the specimens as close as possible to the intact condition. This approach was refined through previous experiments [23], and the parameters were applied identically to all specimen groups.

### 2.5 Measurement of Flexibility and Disc Height Change

Flexibility was assessed in the intact condition on day 0, after incubation on day 7, after loading on day 7, and post‐injection of hydrogel on day 7 (Figure 2A). ROM and NZ were measured under quasi‐static conditions in three anatomical planes: flexion–extension (FE), lateral bending (LB), and axial rotation (AR), following widely accepted testing standards using a universal spine tester (Figure 2C) [39, 40, 43, 44]. A total of 3.5 cycles of pure moments of ±1 Nm were applied at a speed of 1.0°/s without preload, as previously described for bovine tail segments [23]. Motions in terms of ROM and NZ were tracked using a motion tracking system (Vicon Nexus 1.4.116, Vicon Motion Systems Ltd., Oxford, UK) with six cameras (Type MX13, Vicon Motion Systems Ltd.). To minimize viscoelastic effects, two loading cycles were applied for preconditioning, and the third cycle was used to evaluate ROM and NZ. For each motion plane, the total ROM and NZ were calculated as the sum of the ranges on both sides. To enable better comparison within each group, ROM and NZ values were normalized to the intact ROM of each individual specimen (from day 0, except group fresh, from day 7).

Disc height change was measured in the intact condition on day 0, after incubation on day 7, after loading on day 7, and post‐injection of hydrogel on day 7. The height was determined using a universal testing machine (Z01, Zwick Roell, Ulm, Germany) under a low compression force at a defined loading plateau of 30 N (Figure 2D) [23]. The change in disc height was defined as the difference between the initially intact measured value and the value measured in all subsequent conditions. The absolute intact disc height was determined using *n* = 10 representative, sagittal and calibrated radiographs.

### 2.6 Hydrogel Preparation and Injection

The hydrogel components were thawed on a roller mixer (20 rpm, room temperature) for 40 min according to the manufacturer's instructions, and then filled bubble‐free into double‐chamber syringes for injection (Albugel, TETEC AG, Reutlingen, Germany). The hydrogel consisted of a main component containing primarily serum albumin and hyaluronic acid, which was specially modified for this experiment by adding 100 mg/mL of radiopaque aluminum oxide. The second component was a bis‐thio‐polyethylene‐glycol‐HCl crosslinker. The proper solidification of the gel was pre‐tested beforehand by the manufacturer and monitored in each experiment using a control quantity. Furthermore, changes in the viscoelasticity of the gel due to aluminum oxide particles were taken into account during development, and sedimentation of the particles was prevented.

Injection was performed using a 27 G needle into the three‐dimensional center of the IVD. First, the needle was inserted freehand into the center of the disc, and its position was verified via sagittal X‐ray. If needle repositioning was necessary, it was performed only within the existing insertion hole through minor adjustments. As soon as the position was correct, the hydrogel was injected. To determine the maximum injectable volume for each model, the hydrogel was injected until no further volume could be injected. The injected volume was measured by reading the syringe scale.

### 2.7 Hydrogel Distribution Analysis Using microCT

Depending on the specimen, either biomechanical tests (ROM, NZ, disc height) (*n* = 6) or exemplary microCT scans (*n* = 2) were performed (Bruker Skyscan 1172, Bruker Corporation, Billerica, MA). The entire specimens were directly scanned at the day of injection (day 7) without modifications (no freezing or testing). The specimens were mounted on a mounting tray with playdough (Hasbro Inc., Pawtucket, RI) and covered with a radiolucent plastic hood to prevent drying out of the specimen. The capture area of the detector was aligned to the height range of the IVD. Scanning was performed using a resolution of 8.68 μm, rotation steps of 0.3°, a peak voltage of 50 kV, and a source current of 200 μA. After the scans of about 4 h, the raw images were aligned and the model was reconstructed within a Hounsfield scale of −1769 to 974 HU (NRecon software, Bruker Corporation). The visualization was performed with the CTvox software (Bruker Corporation). For coloring, green was assigned to 27–109 HU and red to 152–175 HU.

### 2.8 Macroscopic Appearance

Following all tests, the segments were cut in sagittal direction, and photos were taken. Macroscopic changes were determined by transverse dissection of the IVD near the cranial endplate. No further tests were carried out on the specimens. Macroscopic appearance no. 1 was performed after the biomechanical tests (ROM, NZ, disc height) that followed incubation and complex loading (Figure 2A). Macroscopic appearance no. 2 was performed after hydrogel injection and subsequent biomechanical testing (ROM, NZ, disc height). A representative example of the six dissected discs per group is reported (Figure 6).

### 2.9 Data Analysis and Statistics

Data were collected and processed using Microsoft Excel 16.872024 (Microsoft Corp., Redmond, WA, USA). Statistical analysis and diagrams were carried out using IBM SPSS Statistics Version 29 (IBM Corp., Armonk, NY, USA) and GraphPad Prism 10 (GraphPad Software Inc., Boston, MA, USA). The Shapiro–Wilk test was used to assess the normality of all data and tests confirmed the normality assumption in 81.9% of all subgroups for ROM and NZ, in 100.0% for DH, and in 75.0% for the injected volume, respectively. Due to small sample numbers, non‐parametric tests were used and median values are reported. Differences between groups (fresh, chABC, sham, and papain) within each condition were compared using the Kruskal–Wallis test, followed by pair‐wise Mann–Whitney *U* tests and correction for multiple comparisons [45]. For the fresh group, Wilcoxon signed‐rank test was used to determine differences between the “intact” and the “after hydrogel injection” conditions. For all other groups, the Friedman test with paired Bonferroni–Holm correction was performed to determine the differences between the conditions. All tests were performed with a significance level set to *α* = 0.05.

## 3 Results

### 3.1 Flexibility

**FIGURE 3:**
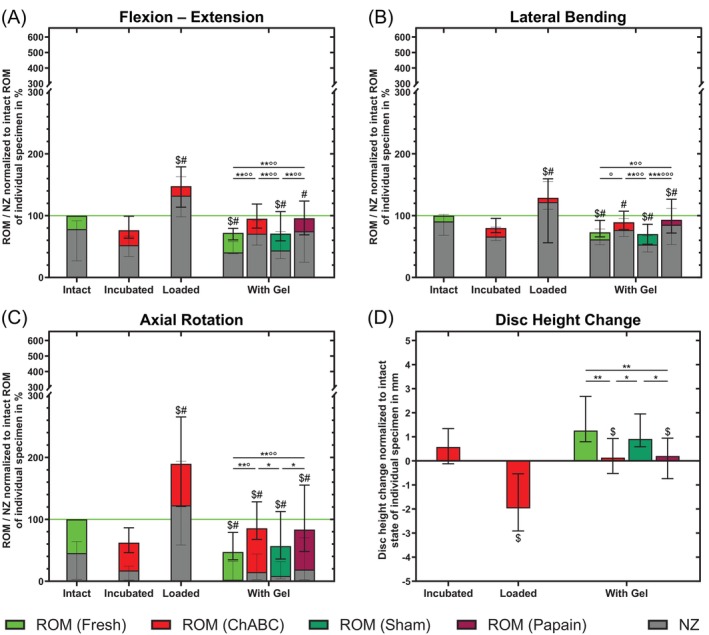
(A–C) Flexibility in three motion planes and (D) disc height change for different conditions. Columns show the median range of motion (ROM) or disc height change in colors with minimum and maximum values as bold error bars. Neutral zone (NZ) is represented by gray columns and thin error bars for minimum and maximum. Individual sample size numbers (at least *n* = 6 per group) can be obtained from Figure 2. Significant differences for a certain condition compared to the previous condition within a group are indicated by $ for ROM or disc height and # for NZ: $ *p* < 0.05, # *p* < 0.05. Significant differences between the groups at certain conditions are indicated by * for ROM or disc height and ° for NZ: * *p* < 0.05, ** *p* < 0.01, *** *p* < 0.001, ° *p* < 0.05, °° *p* < 0.01, °°° *p* < 0.001.

In the intact condition, the ROM was 100% due to normalization in all principal motion planes and the NZ for FE 78.1%, for LB 90.6%, and for AR 45.5%. With heterogeneity between the individual specimens, during incubation, ROM decreased for chABC to 76.5% in FE (*p* = 0.114), to 80.1% in LB (*p* = 0.102), and to 62.5% in AR (*p* = 0.068) (Figure 3A–C). After complex loading, an increase of 148.3% for FE, 130.8% for LB, and 192.1% for AR was observed (*p* ≤ 0.020). Likewise for NZ, a decreasing trend could be observed for some specimens in all three motion planes after incubation (*p* ≤ 0.188) and an increase after complex loading (*p* ≤ 0.004). The injection of the hydrogel increased the ROM and the NZ compared to the previous condition in all groups, but only slightly for chABC (ROM: *p* ≤ 0.114; NZ: *p* ≤ 0.102): The ROM after hydrogel injection for chABC was 95.0% in FE, 89.3% in LB, and 85.5% in AR as well as the NZ was 70.9%, 76.9%, and 14.7%, respectively. Thereby, the ROM and the NZ for chABC were restored by the hydrogel in comparison to the intact condition for all three motion planes (ROM: *p* ≥ 0.219; NZ: *p* ≥ 0.188). After hydrogel injection, no differences were observable between the chABC and papain treated groups (ROM: *p* ≥ 0.568; NZ: *p* ≥ 0.348). With regard to the control groups, ROM did not differ after hydrogel injection between fresh and sham (*p* ≥ 0.289), and the same was observed for NZ—in AR: *p* = 0.094, otherwise *p* ≥ 0.391. Additionally, for ROM and NZ, chABC and papain versus fresh and sham formed two distinct flexibility ranges (*p* ≤ 0.041) with the exception of fresh/chABC for ROM in LB (*p* = 0.086) and sham/chABC and sham/papain for NZ in AR (*p* ≥ 0.165).

### 3.2 Disc Height Change

Treatment with chABC and subsequent complex loading, decreased the disc height by 2.1 mm (*p* = 0.012) (Figure 3D). With a disc height change of only 0.1 mm for chABC and 0.3 mm for papain, the injection of hydrogel restored the height for the enzyme‐treated groups (*p* ≥ 0.564). For the condition “after hydrogel injection”, chABC and papain versus fresh and sham formed two distinct disc height ranges (*p* ≤ 0.041) meaning there were no differences between chABC and papain (*p* = 0.683) as well as between fresh and sham (*p* = 0.462). The initial disc height from calibrated radiographs was 6.9 mm (5.4–8.1 mm range).

### 3.3 Injectable Volume

**FIGURE 4:**
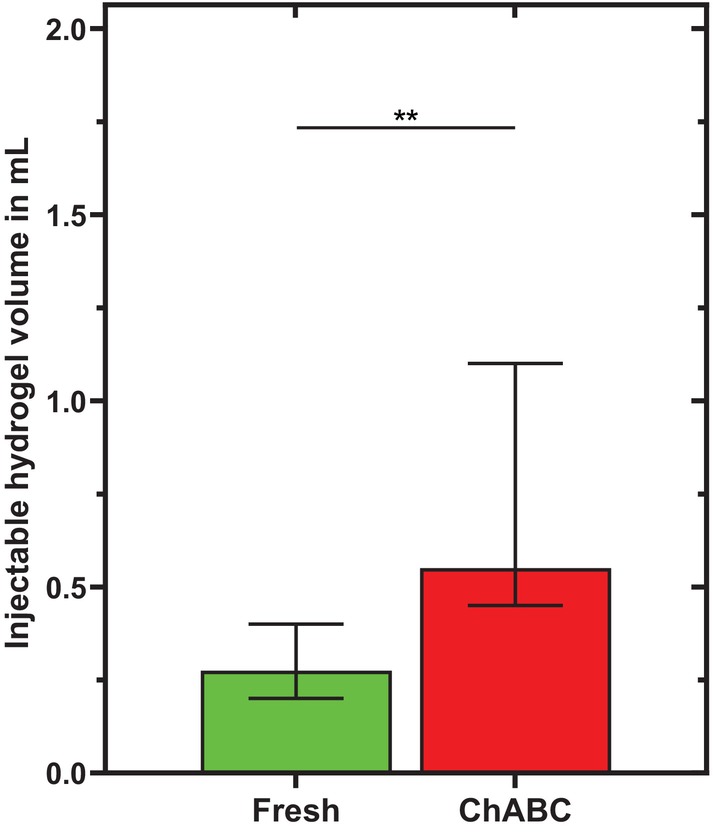
Maximal injectable hydrogel volume in mL for discs of all four groups (*n* = 8). * highlight significant differences between the groups: ** *p* < 0.01.

IVDs treated with chABC allowed more hydrogel injection (0.6 mL; 0.5–1.1 mL) than fresh IVDs (0.3 mL; 0.1–0.7 mL) (*p* = 0.002, Figure 4).

### 3.4 Hydrogel Distribution

**FIGURE 5:**
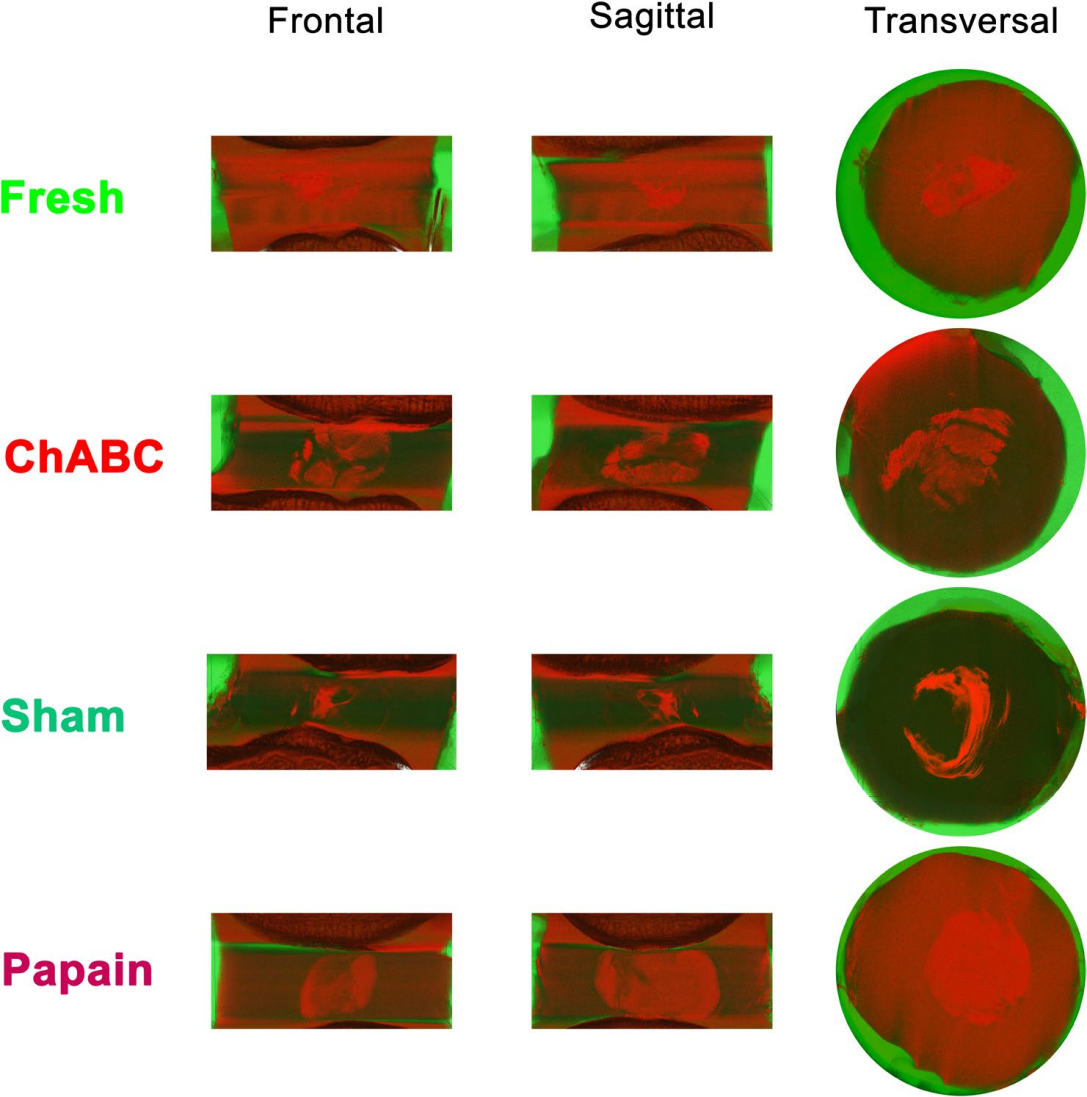
Hydrogel distribution in three different view directions—frontal, sagittal, and transversal—for one exemplary motion segment (Bone—Disc—Bone) per group. Hydrogel with radiopaque aluminum oxide particles was injected into the disc center in both treated (chABC, sham, papain) and untreated (fresh) conditions. MicroCT was used to visualize the segment, including the disc, hydrogel, and adjacent visible vertebrae (red coloration). Green coloration represents empty scan chamber volume (air). In the transverse view, the embedding material PMMA is also visible.

MicroCT imaging allowed the visualization of bone, IVD, and hydrogel as exemplary observations (Figure 5). The coloring was based on HU values and differentiated the environment (air) in shades of green from the specimen structures (bone, IVD and hydrogel) in shades of red. For fresh, small areas of hydrogel appeared in the center of the disc. ChABC was distributed in a bubble‐like (also cumulus‐like) pattern with darker colored interspaces. The hydrogel in the sham group was similar to the pattern of the fresh group in the frontal and sagittal views, but the transverse layer showed that the hydrogel could also have been distributed in a larger ring shape. In the papain group, the hydrogel formed a well‐defined sphere. Furthermore, the hydrogel volume in chABC and papain groups appeared to be larger than the volumes of the fresh and sham groups, corresponding to the higher injection volume.

### 3.5 Macroscopic Appearance

**FIGURE 6:**
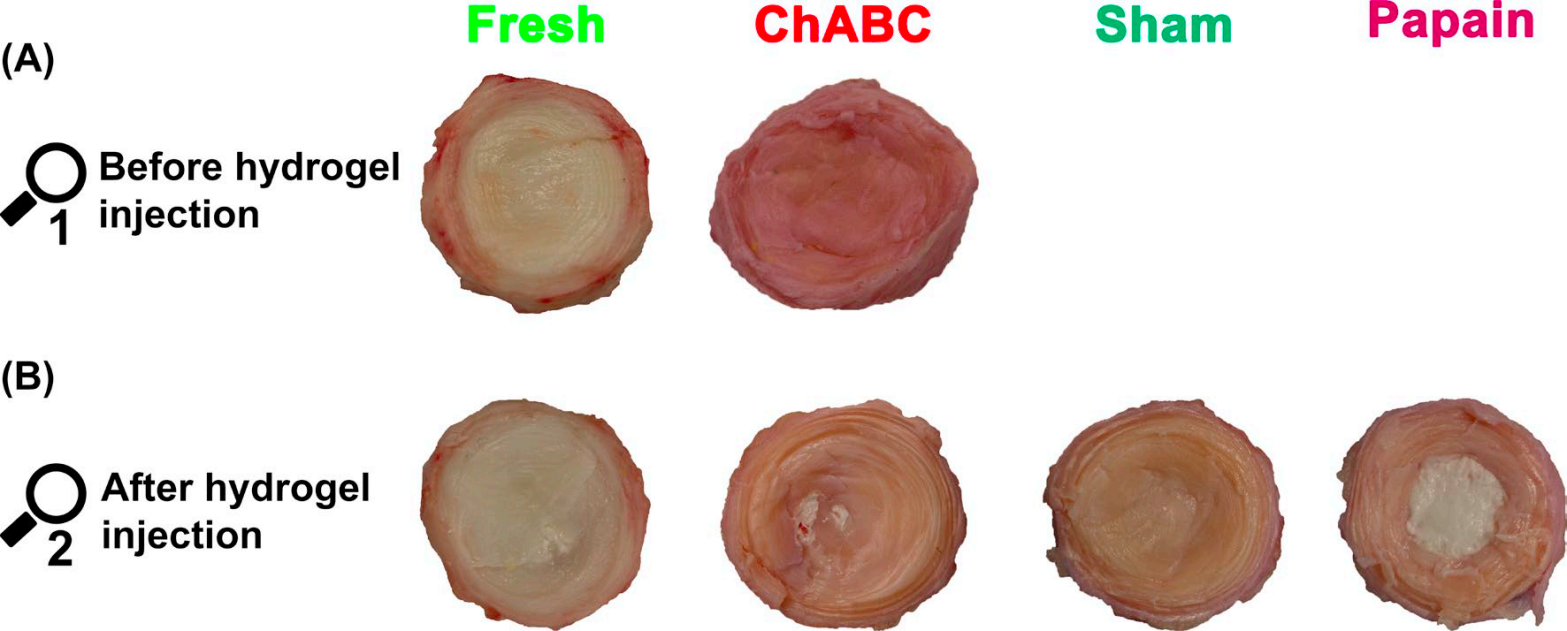
Macroscopic appearance of one exemplary disc per group: (A) before hydrogel injection. (B) After hydrogel injection. For sham and papain before hydrogel injection, please refer to the previously published images [23]. Further images can be found in Table S1.

Discs treated with chABC for 7 days, as well as untreated fresh discs, appeared macroscopically unchanged (Figure 6A). No cavities, fissures, clefts, or visible defects were observed. The injected hydrogel (white in appearance due to aluminum oxide) was visible in all groups after injection. For fresh, chABC, and sham, hydrogel could only be found in a few tiny areas, but for papain, a well‐confined hydrogel core was visible in the center of each disc (Figure 6B and Table S1).

## 4 Discussion

This study successfully quantified the biomechanical effects of 5 U/mL chABC on a segmental level for bovine tail specimens and found an increase for ROM, NZ, and injectable volume, along with a simultaneous decrease in disc height after 7 days of treatment. Compared to 65 U/mL papain, the most striking finding is that both enzymes cause nearly identical biomechanical changes (in magnitudes), but macroscopically, chABC‐treated IVDs remain intact, whereas papain‐treated ones show a cavity [23]. Consequently, the injected hydrogel is distributed in a bubble‐like pattern for chABC, and spherically for papain. The methods used have allowed the acquisition of consistent results that confirm previous studies, and it can be concluded that the findings support two different preclinical models: chABC as model for moderate degeneration and papain as a model for nucleotomy.

Such models are increasingly needed as injectable therapies or nucleus replacements continue to be developed, and bovine tail segments are more frequently used for in vitro testing [17, 19, 20, 23, 46, 47, 48, 49, 50]. It has become clear that injecting biomaterials into fresh IVDs from young animals is technically difficult—also confirmed by the fresh group, where only 0.1 mL of hydrogel could be injected (Figure 4) [51]. Moreover, animal IVDs often show no signs of degeneration and thus do not represent the clinical condition of patients eligible for disc therapies [23, 24, 25]. Performing a mechanical nucleotomy is also disadvantageous for biomechanical tests due to severe annulus damage [25, 52]. Therefore, enzymatic treatments are beneficial, as their degradative effects allow biomaterial injections into the NP (here, up to 0.6 mL for chABC), leaving the AF mostly intact and avoiding the need for mechanical nucleotomy, while also promoting degenerative effects on the cellular and biochemical level [28].

While papain was shown to create a cavity, the disc tissue remained macroscopically unchanged during chABC treatment, a finding widely reported by other authors [23, 24, 28, 51]. This can be explained by chABC's specific digestion of GAGs, which occurs in a dose‐ and time‐dependent manner, with less severe damage to the extracellular matrix than papain [24, 33, 53]. This leads to depressurization in the NP and progressive disc degeneration without a cavity formation. For biomechanical comparison, ROM, NZ, disc height change, and injectable volume are summarized in tabular form (Tables 1, 2, 3, 4), which also include results from our previous experiments with papain [23]. New statistical comparison of the previous data versus that from the current study reveals small differences between chABC and papain in ROM and NZ after incubation; however, these differences fully disappear after complex loading (ROM: *p* ≥ 0.583; NZ: *p* ≥ 0.305). Disc height changes after incubation (*p* = 0.145) and after loading (*p* = 0.787) are similar for both enzymes and significantly different from the sham group (*p* ≤ 0.002). These results confirm that both enzyme models increase flexibility and instability while reducing disc height [23, 28, 31, 32, 35, 37, 51, 54]. The same amount of hydrogel could be injected within both enzyme‐treated groups (chABC and papain; *p* ≥ 0.520), whereas significantly less—but still comparable amounts—were injectable into the fresh and sham groups (*p* ≥ 0.999), with the two sets of groups differing significantly from each other (Table 4; *p* ≤ 0.002).

**TABLE 1:** Comparison of ROM between the groups chABC, fresh, papain, and sham.

Motion plane	Group	Intact	After incubation	After loading	After injection
Flexion–extension	Fresh	100.0			69.0 (60.8–75.2)
	ChABC	100.0	76.5 (63.6–99.0)	148.3 (120.3–163.1)	95.0 (79.9–118.7)
	Sham	100.0	62.9 (48.6–75.7)^a^	126.7 (98.6–155.3)^a^	70.9 (59.1–106.6)
	Papain	100.0	86.8 (66.6–139.1)^a^	144.8 (115.5–218.0)^a^	95.7 (81.1–113.4)
Lateral bending	Fresh	100.0			72.6 (65.7–92.4)
	ChABC	100.0	80.1 (73.3–95.6)	130.8 (56.1–159.5)	89.3 (77.3–107.2)
	Sham	100.0	65.7 (51.0–82.8)^a^	116.1 (92.5–139.4)^a^	68.7 (53.7–76.9)
	Papain	100.0	92.1 (67.2–137.3)^a^	125.8 (98.7–188.8)^a^	93.3 (86.3–107.4)
Axial rotation	Fresh	100.0			46.5 (34.5–68.9)
	ChABC	100.0	62.5 (55.0–86.4)	192.1 (153.7–265.4)	85.5 (67.6–128.2)
	Sham	100.0	50.5 (27.5–72.1)^a^	137.8 (92.9–227.0)^a^	56.2 (35.9–73.1)
	Papain	100.0	78.8 (62.5–201.9)^a^	202.9 (126.2–469.7)^a^	86.6 (67.4–155.3)

*Note:* Total ROM is normalized to the intact condition in % and shown as median values (min–max) for the three principal motion planes at ±1 Nm: Intact, after incubation, after loading, after hydrogel injection. Sample size per group *n* ≥ 6; for more details please refer to Figure 2A.

^a^ Usage of data from Jansen et al. [23].

**TABLE 2:** Comparison of NZ between the groups chABC, fresh, papain, and sham.

Motion plane	Group	Intact	After incubation	After loading	After injection
Flexion–extension	Fresh	78.1 (26.7–91.6)			39.4 (38.3–45.3)
	ChABC	78.1 (26.7–91.6)	52.8 (43.1–67.0)	133.9 (120.3–163.1)	70.9 (52.3–91.7)
	Sham	78.1 (26.7–91.6)^a^	33.7 (26.9–40.3)^a^	109.7 (87.1–139.7)^a^	43.4 (30.5–47.7)
	Papain	78.1 (26.7–91.6)^a^	63.1 (46.9–110.4)^a^	126.0 (40.0–201.8)^a^	75.8 (58.0–90.2)
Lateral bending	Fresh	90.6 (68.1–102.1)			60.5 (52.8–77.0)
	ChABC	90.6 (68.1–102.1)	68.5 (63.6–81.4)	124.9 (118.6–155.0)	76.9 (66.2–95.5)
	Sham	90.6 (68.1–102.1)^a^	46.8 (36.7–62.4)^a^	111.7 (89.7–133.0)^a^	51.9 (41.3–64.2)
	Papain	90.6 (68.1–102.1)^a^	80.9 (68.7–127.4)^a^	120.8 (87.1–186.1)^a^	85.3 (77.7–99.5)
Axial rotation	Fresh	45.5 (3.1–64.1)			1.9 (0.3–12.8)
	ChABC	45.5 (3.1–64.1)	18.3 (3.6–24.2)	124.9 (98.6–193.8)	14.7 (2.7–44.0)
	Sham	45.5 (3.1–64.1)^a^	5.6 (1.6–16.0)^a^	87.3 (56.1–154.1)^a^	8.3 (3.8–23.5)
	Papain	45.5 (3.1–64.1)^a^	27.0 (0.9–112.9)^a^	136.6 (4.9–354.1)^a^	24.4 (5.3–42.4)

*Note:* Total NZ is normalized to the intact ROM in % and shown as median values (min–max) for the three principal motion planes at ±1 Nm: Intact, after incubation, after loading, after hydrogel injection. Sample size per group *n* ≥ 6; for more details please refer to Figure 2A.

^a^ Usage of data from Jansen et al. [23].

**TABLE 3:** Comparison of disc height change between the groups chABC, fresh, papain, and sham.

Group	After incubation	After loading	After injection
Fresh			1.4 (0.8/2.7)
ChABC	0.4 (−0.1/1.3)	−2.1 (−2.9/−1.2)	0.1 (−0.5/0.9)
Sham	1.3 (0.5/2.5)^a^	−0.6 (−1.0/0.1)^a^	0.9 (0.6/1.9)
Papain	0.2 (−0.5/0.6)^a^	−2.2 (−3.1/−1.7)^a^	0.3 (−0.6/0.7)

*Note:* Disc height change is normalized to intact height in mm and shown as median values (min/max) at a compression load of 30 N: After incubation, after loading, after hydrogel injection. Sample size per group *n* ≥ 6; for more details please refer to Figure 2A.

^a^ Usage of data from Jansen et al. [23].

**TABLE 4:** Comparison of maximal injectable hydrogel volume between the groups chABC, fresh, papain, and sham.

Group	After injection
Fresh	0.3 (0.1–0.7)
ChABC	0.6 (0.5–1.1)
Sham	0.3 (0.2–0.4)^a^
Papain	0.8 (0.5–1.5)^a^

*Note:* Maximal injectable hydrogel volume in mL is shown as median values (min–max). Sample size per group *n* = 8.

^a^ Usage of data from Jansen et al. [23].

ROM and NZ as metrics for flexibility and instability are important parameters for the evaluation of spinal therapies. Together with the study on the enzyme papain, we provide the first ROM and NZ data in the literature on bovine tail FSUs [23]. ROM and NZ show a clear inverse relationship to the disc height in all four groups. After incubation, the increase in disc height and the stiffening effect can be attributed to the fluid uptake of the disc and a subsequent re‐orientation of AF fibers to a more axial direction [23, 34, 55, 56, 57, 58, 59, 60]. However, the enzymes weaken the stiffening effect compared to sham, probably due to matrix degradation processes—specifically, glycosaminoglycan (GAG) degradation in the case of chondroitinase ABC (chABC), and a broader, unspecific proteolysis of structural proteins in the case of papain [23, 28, 31, 38, 61]. These distinct biochemical mechanisms likely explain the observed differences in tissue response: chABC degrades proteoglycans by cleaving glycosaminoglycan chains, closely resembling natural degeneration processes and possibly leading to a more physiologically relevant distribution pattern within the matrix. In contrast, papain, a proteolytic enzyme with broad substrate specificity—commonly used in food processing to tenderize meat—indiscriminately breaks down various proteins, which may cause less specific structural alterations [23, 28, 31, 38, 61]. The effect is stronger for papain than for chABC in the LB and AR motion plane (Figure 3). After loading, the effects of chABC and papain become even more clearly visible. As already measured for rabbits, monkeys and dogs in the 1990s [31, 36, 37, 54], the disc height decreases with a simultaneous increase in ROM and NZ. In this context, it is worth emphasizing that, the ROM results that we measured for chABC‐treated bovine tail segments (1 U/disc, 7 days), quantitatively agree well with the results obtained by Lu et al. with in vivo‐treated dog specimens (5 U/disc, 1 week): For FE 140% (Lu et al.) vs. 148.3%, for LB 129% (Lu et al.) vs. 130.8%, and for AR 150% (Lu et al.) vs. 192.1%. The fact that higher chABC concentrations are required in vivo could be due to the different species and nucleus sizes or the organism's reaction against the enzyme, which was already observed for papain by Potter et al. [33, 62]. Additionally, in vivo, discs are dynamically loaded and diurnal loading has been shown to change degradation dynamics for chABC, potentially due to a difference in molecular transport [33, 63]. In respect of disc height, both enzymes caused a similar loss in height of 2.1 and 2.2 mm which resulted in a percentual height reduction of between 30% and 32% based on an initial disc height of 6.9 mm. According to the disc degeneration grading for the lumbar spine from Wilke et al., this corresponds to a mild degeneration (< 33%) on the edge of a moderate degeneration (≥ 33%) [22].

The increased flexibility in both models is contrary to natural disc degeneration [64, 65, 66, 67] but most likely also due to the high enzyme concentrations. The concentration of 5 U/mL chABC used here is one of the highest concentrations reported in the literature. There are two different frequently used concentration levels in the literature: Some studies use concentrations in the range of 0.25 U/mL [34, 68, 69, 70] and others use concentrations in the range of 5 U/mL [32, 33, 53, 71, 72]. After we found no differences in an initial study with 0.25 U/mL chABC compared to sham‐treated discs, we switched to the other often reported concentration of 5 U/mL. Interestingly, 5 U/mL of chABC caused strong biomechanical changes with the same magnitude as papain.

Macroscopic images [52, 73], microscopy [50], X‐ray [23], or MRI [74], have been used in previous studies to attempt to visualize the injected biomaterials in the IVD. Similar to Gullbrand et al., Muir et al., and Wilke et al., we use microCT to visualize the radiopaque hydrogel within the disc [72, 75, 76]. Generally, it is visually clear that less volume has been injected into the control groups than into the enzyme‐treated groups (Figure 4 vs. Figure 5). In chABC‐treated IVDs, it can be observed that the hydrogel has spread in a bubble‐like shape with multiple hydrogel pockets (sagittal view) and that it has widely distributed between annular layers of the inner AF (transversal view) (Figure 5). Regardless of chABC's effects on the AF, we hypothesize that such a distribution between the layers is only possible with an initially liquid hydrogel, as was the case with our hydrogel. Additionally, the hydrogel distribution for chABC corresponds very closely to the transverse microCT visualization of Gullbrand et al. who also simulated disc degeneration with 1 U/disc of chABC [72]. With regard to suitability of chABC as a disc degeneration model, it must be emphasized that the bubble‐like distribution matches more closely the X‐ray images of discographs in patients with degenerated disc than with papain treatment [77]. The hydrogel in papain‐treated IVDs can be identified as a monolithic, spherical mass in the center of the IVDs (Figure 5). Comparable patterns are documented in the literature only with transversal macroscopic images (Rahman et al.: hydrogel B) [48]. These images are interestingly based on nucleotomies performed on bovine tail discs, hence, supporting the hypothesis that papain treatment simulates a nucleotomy [48].

Limitations of the study include the microCT scans, which were performed on only two specimens per group as exemplary observations rather than a full dataset. The scans required several hours and the specimens had to remain unfrozen and unavailable for subsequent biomechanical testing—the main focus of the study, so increasing the number of samples was not feasible. However, when considered together with the other results of this study and the relevant literature, their interpretive value is strengthened [23, 24, 28, 33, 35, 48, 51, 72, 76, 77]. The use of bovine tail segments introduces additional limitations regarding transferability to the human spine, as these specimens differ in anatomy, size, geometry and shape, absence of facet joints, and presumed physiological loading condition [19, 20, 78]. Nonetheless, they offer good uniformity, minimal ethical concerns, easy availability and their biological and biomechanical properties are well researched [23, 28, 33, 35, 48, 51, 74, 79, 80]. This study shows that the bovine tail in this PMMA embedded organ culture approach is well suited as a model for injectable therapies—for both chABC and papain [23]. Further limitations relate to the injection procedure. The volume of hydrogel injected was not predefined; instead, the aim was to determine the maximum amount that could be delivered, while injection pressure could not be quantified, limiting standardization. Radiopaque particles added to the hydrogel may have influenced rheology, although the particle size was very small and the gel has been injectable via a 27 G needle (0.36 mm). However, the reason why only small needles (30 G for enzymes; 27 G for gel) were used is that AF injury became a controversial topic after Carragee et al. showed the promotion of disc degeneration by injections into the disc, such as by discography with needle sizes of 22–25 G [81]. In contrast, Elliott et al. described that for a needle‐size‐to‐disc‐height ratio below 25% (given for 27 G and bovine discs) the needle injury has no effect, such as on the NP depressurization [81, 82].

All in all, both models provide arguments for their use as disc degeneration models for different applications. There are stronger arguments that chABC simulates disc degeneration better than Papain. Papain, on the other hand, especially at high concentrations, better mimics a model for nucleotomies. Overall, both models offer possibilities for further development and modification, making them very flexible and adaptable testing platforms. Future studies may adapt the dose and exposure time of the enzymes to individual requirements to achieve a suitable balance of ROM, NZ and disc height changes and injectable biomaterial volume. As the specimens are already embedded for biomechanical testing, further biomechanical tests can easily follow the injection of biomaterials. Furthermore, complex dynamic loading could be used as a dynamic test method to determine the extrusion risk of biomaterials. Also feasible, the intact AF could be opened with a standardized defect for testing annulus closure techniques. In addition, the models enable a combined investigation of biomechanical and biological aspects, as the models demonstrate a sterile organ culture period. Thus, in a collaborative project with Vernengo et al. the same methods as in this work have been used to investigate the cellular, biochemical and histological properties in the AF after enzyme treatment [28]. The results of the studies confirm each other and additional findings on matrix composition, cell viability, and gene expression have been demonstrated [28]. This also permits, for example, the combination of both models with growth factors, hormones, cell therapies and the evaluation of biological parameters and interactions such as the interaction of injected biomaterials with the IVD tissue.

## 5 Conclusion

ChABC leads to a strong increase in flexibility and instability of bovine tail segments, reduces disc height and allows the injection of hydrogels into the disc (median volume 0.6 mL). The nucleus remains macroscopically intact. ChABC produces similar biomechanical effects on the same magnitude compared to the enzyme papain (Tables 1, 2, 3, 4). However, regarding hydrogel distribution, cavity formation and other reports in the literature, it can be concluded that chABC better simulates disc degeneration whereas papain produces similar effects to a nucleotomy. Both models allow advanced preclinical testing of biomaterial and can be further developed for dynamic testing (e.g., to test the extrusion risk of biomaterials).

## Author Contributions

All authors contributed to the study conception and design. Jan Ulrich Jansen developed the methods, carried out the studies, and conducted the data analysis. Jan Ulrich Jansen, Graciosa Quelhas Teixeira, Cornelia Neidlinger‐Wilke, and Hans‐Joachim Wilke conceived the studies. Elias Salzer, Andrea Vernengo, Sibylle Grad, and Keita Ito participated in developing the organ culture model with Chondroitinase ABC. The first draft of the manuscript was written by Jan Ulrich Jansen, and all authors commented on previous versions of the manuscript. All authors read and approved the final manuscript.

## Funding

This work was supported by European Union's Horizon 2020 research and innovation program iPSpine, Grant/Award Number: 825925.

## Ethics Statement

The authors have nothing to report.

## Conflicts of Interest

The authors declare no conflicts of interest. Sibylle Grad is an Editorial Board member of JOR Spine. She was excluded from editorial decision‐making related to the acceptance of this article for publication in the journal.

## Supporting information


**Appendix S1:** Supplementary information.

## Data Availability

The data that support the findings of this study are available from the corresponding author upon reasonable request.
