# Supplementary material for: Chondroitinase Versus Papain Digestion Leads to Different Outcome for In Vitro Simulation of Degenerated Discs
Source: JOR Spine. 2026 Mar 1;9(1):e70164. doi: 10.1002/jsp2.70164 (PMC12950828; doi:10.1002/jsp2.70164)
Supplement: Supplementary file 1 — Appendix S1: Supplementary information. [file JSP2-9-e70164-s001.docx]

TABLE S1 Additional macroscopic images of two more exemplary discs per group: A, before hydrogel injection. B, after hydrogel injection. For sham and papain before hydrogel injection, please refer to the previously published images (Jansen et al., 2024).

| **Time point** | **Set** | **Fresh** | | **ChABC** | | **Sham** | | **Papain** | |
| --- | --- | --- | --- | --- | --- | --- | --- | --- | --- |
| **(A) Before hydrogel injection** | **2** | 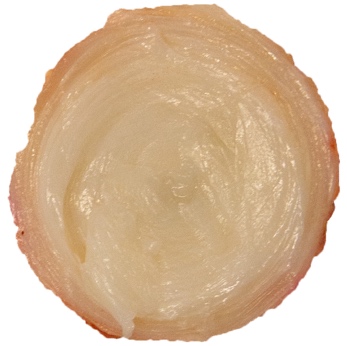 | 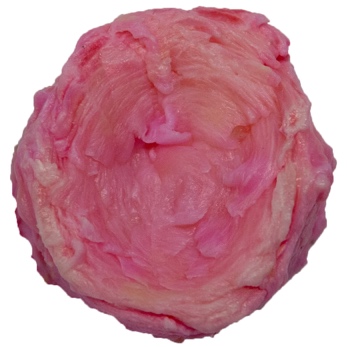 | |  | |  | |  |
|  | **3** | 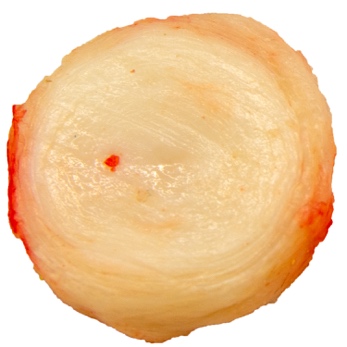 | 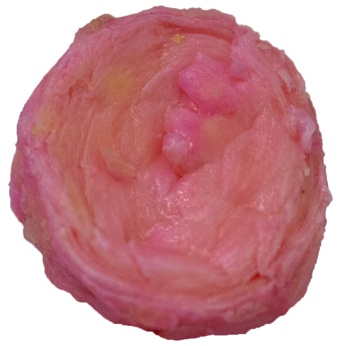 | |  | |  | |  |
| **(B) After hydrogel injection** | **2** | 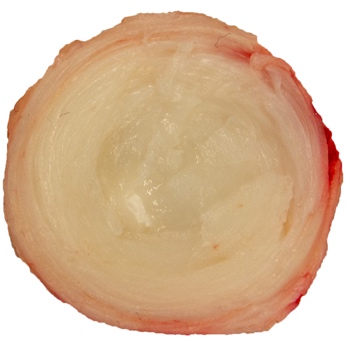 | 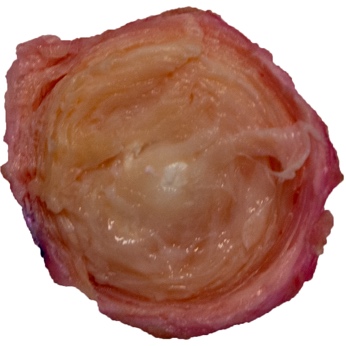 | | 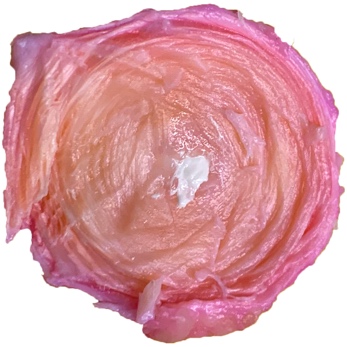 | | 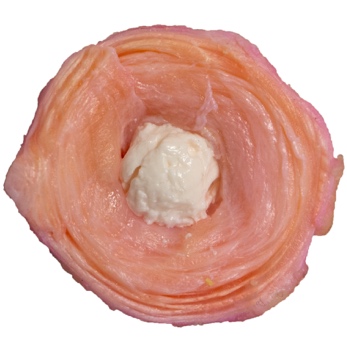 | |  |
|  | **3** | 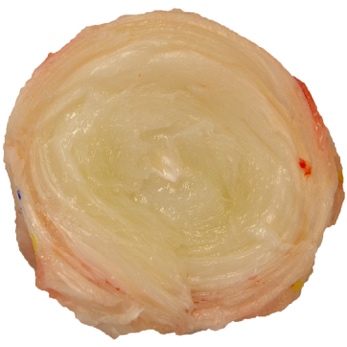 | 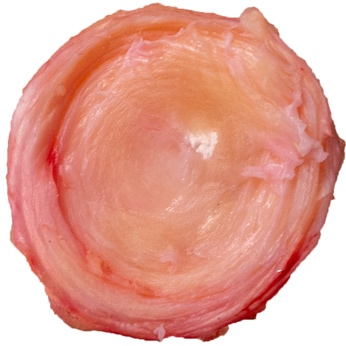 | | 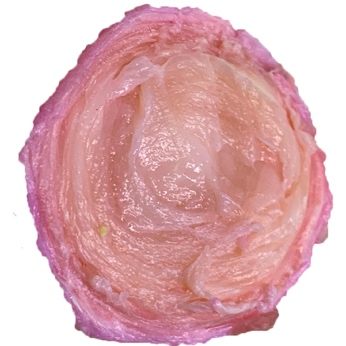 | | 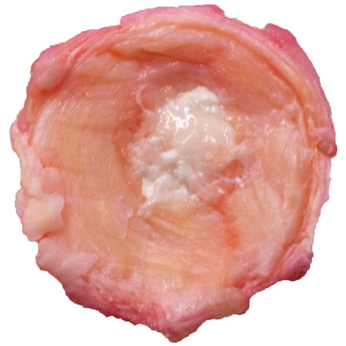 | |  |
